# Effect of S267F variant of NTCP on the patients with chronic hepatitis B

**DOI:** 10.1038/s41598-017-17959-x

**Published:** 2017-12-15

**Authors:** Hye Won Lee, Hye Jung Park, Bora Jin, Mehrangiz Dezhbord, Do Young Kim, Kwang-Hyub Han, Wang-Shick Ryu, Seungtaek Kim, Sang Hoon Ahn

**Affiliations:** 1 Department of Internal Medicine, Institute of Gastroenterology, Yonsei University College of Medicine, Seoul, Korea; 2 Department of Biotechnology, Yonsei University, Seoul, Korea; 3 Severance Biomedical Science Institute, Yonsei University College of Medicine, Seoul, Korea; 4 Department of Biochemistry, Yonsei University, Seoul, Korea; 5 Institut Pasteur Korea, Seongnam-si, Gyeonggi-do Korea

## Abstract

Sodium taurocholate cotransporting polypeptide (NTCP) was identified as an entry receptor for hepatitis B virus (HBV) infection. The substitution of serine at position 267 of NTCP with phenylalanine (S267F) is an Asian-specific variation that hampers HBV entry *in vitro*. In this study, we aimed to evaluate the prevalence of S267F polymorphism in Korean patients with chronic hepatitis B (CHB) and its association with disease progression and potential viral evolution in the preS1 domain of HBV. We found that the frequency of the S267F variant of NTCP in CHB patients and controls was 2.7% and 5.7% (*P* = 0.031), respectively, and that those who had S267F variant were less susceptible to chronic HBV infection. The frequency of the S267F variant in CHB, cirrhosis and hepatocellular carcinoma (HCC) patients was 3.3%, 0.9%, and 3.5%, respectively. Thus, the S267F variant correlated significantly with a lower risk for cirrhosis (*P* = 0.036). Sequencing preS1 domain of HBV from the patients who had S267F variant revealed no significant sequence change compared to the wild type. In conclusion, the S267F variant of NTCP is clinically associated with a lower risk of chronic HBV infection and cirrhosis development, which implicates suppressing HBV entry could reduce the disease burden.

## Introduction

Chronic hepatitis B virus (HBV) infection is a leading cause of cirrhosis and hepatocellular carcinoma (HCC)^1^ and a high level of HBV DNA is known as an independent risk factor for the development of cirrhosis and HCC^2–4^. Thus, eradication or prolonged suppression of HBV replication could, in principle, reduce the risk of liver disease progression. Currently, elimination of the virus (“cure”) is not achievable but suppression of viral genome replication has been quite successful since the introduction of nucleos(t)ide analogues such as entecavir or tenoforvir.

HBV, a causative agent of the above liver diseases, is an enveloped DNA virus with strict host species and cell type specificity^5^. A complex combination of environmental, pathogenic and host genetic factors plays a role in determining both susceptibility to the virus and the course of the infection. Among these factors, the entry step is vital for establishment of viral infection and this step is generally mediated by interactions between viral envelope glycoprotein(s) and the cellular receptor(s).

The outer membrane of infectious HBV particles contains three envelope glycoproteins: large (L, preS1 + preS2 + S), middle (M, preS2 + S), and small (S only) proteins^6^. Of these, the preS1 domain of L protein is essential for viral attachment to the cellular receptor^7^ and the S domain mediates recruitment of the virus to the hepatocyte surface via heparin sulfate proteoglycans^8,9^. All three proteins (L, M, S) share an identical C-terminal S domain, which contains the hepatitis B surface antigen (HBsAg).

Although the identity of cellular receptor(s) for HBV infection had been a mystery for a long time, sodium taurocholate cotransporting polypeptide (NTCP), a bile acid transporter, was discovered recently as a functional cellular receptor for both HBV and hepatitis delta virus (HDV) infection^7,10^. NTCP protein is encoded by the NTCP *(SLC10A1)* gene and is specifically expressed at the basolateral membrane of hepatocytes as a transmembrane protein. The identification of NTCP as an entry receptor has thus enabled development of *in vitro* cell culture system that allows for HBV infection by overexpressing NTCP protein in hepatoma cells.

Genetic variations can influence the expression and function of proteins. Single nucleotide polymorphisms (SNPs), some of which are specific to certain ethnicities, have been identified in the NTCP gene^11–14^. For example, the genetic variant I223T (c.668 T > C) was identified in African-Americans at a frequency of 5.5%. Other variants, S267F (also known as rs2296651, c.800 C > T, or p.Ser267Phe) and I279T (c.836 T > C), are Asian-specific SNPs, with allele frequencies of 7.5% and 0.5%, respectively. Of these SNPs, S267F is particularly interesting in that it impairs the function of NTCP protein as both bile acid transporter and cellular receptor for HBV infection^10^. Although the effect of this specific SNP was demonstrated in *in vitro* cell culture experiments^15^, the clinical implication of this polymorphism remained to be evaluated.

In this study, we aimed to investigate the frequency of this specific S267F variant of NTCP among Korean patients with chronic hepatitis B and its impact on the natural course of viral infection and potential viral evolution in the preS1 domain of HBV.

## Results

### Baseline characteristics

The baseline characteristics of the 1,376 study subjects are summarized in Table 1. In total, we analyzed 1,200 patients with CHB and 176 controls. For CHB patients, the mean age was 50.5 years, with more males than females (n = 803; 66.9%). Patients with CHB had lower platelet levels and higher AST and ALT levels than controls. The mean HBV DNA level was 2.9 log_10_ IU/mL and 269 (22.4%) patients were positive for HBeAg. In total, 709 (59.1%) patients received antiviral treatment. In the CHB patients, the S267F polymorphism was distributed as follows: 1,168 (97.3%) patients were wild-type homozygotes (CC) and 32 (2.7%) patients were heterozygotes (CT). However, no TT homozygote patient was found.

**Table 1:** Baseline characteristics of the study population.

Variable	CHB (n = 1,200)	Control (n = 176)	*P* value
*Demographic*			
Age, years	50.5 ± 11.7	47.8 ± 13.8	<0.001
Male gender	803 (66.9)	88 (50%)	<0.001
*Laboratory*			
Platelet, 10^3^/μL	92.0 ± 91.2	117.9 ± 104.7	0.001
Serum albumin, g/dL	3.5 ± 0.7	3.6 ± 0.5	0.134
AST, IU/L	122.0 ± 368.9	48.6 ± 53.8	0.043
ALT, IU/L	91.8 ± 169.5	36.8 ± 43.4	0.002
*Polymorphism-related*			
S267F, n (%)			0.031
CC	1,168 (97.3)	166 (94.3)	
CT	32 (2.7)	9 (5.1)	
TT	0 (0)	1 (0.6)	
*HBV-related*			
HBV DNA, log _10_ IU/mL	2.9 ± 2.1		
HBeAg positivity, n (%)	269 (22.4)		
Antiviral therapy, n (%)	709 (59.1)		

Variables are expressed as mean ± SD (range) or n (%). CHB, chronic hepatitis B; AST, aspartate aminotransferase; ALT, alanine aminotransferase; HBV, hepatitis B virus; HBeAg, HBV e antigen.

### Frequency of S267F variant in controls and patients with chronic hepatitis B

Polymorphism distribution analysis showed that the frequency of S267F variant was in Hardy-Weinberg equilibrium. No departure from the Hardy-Weinberg distribution was observed for this polymorphism (*P* = 0.640) in CHB patients or controls.

Notably, the S267F polymorphism was associated with reduced chronic infection in CHB patients compared with controls (Table 2). The distribution of S267F polymorphism differed significantly between CHB patients and controls. The frequency of the S267F variant (CT heterozygote or TT homozygote) in CHB patients was lower than that of controls (2.7% vs. 5.7%). Also, the risk of chronic HBV infection was significantly lower in patients with CT heterozygote than those with CC homozygote (odds ratio [OR] 0.455, 95% confidence interval [CI] 0.220–0.942, *P* = 0.034).

**Table 2:** Association of S267F polymorphism with chronic HBV infection.

Polymorphism	CHB	Controls	OR	95% CI	*P* value
S267F					
CC	1168 (97.3)	166 (94.3)	1.000		
CT	32 (2.7)	9 (5.1)	0.455	0.220–0.942	0.034
TT	0 (0)	1 (0.6)			

Variables are expressed as n (%). CHB, chronic hepatitis B; OR, odds ratio; 95% CI, 95% confidence interval.

### Association of S267F polymorphism with disease course of chronic HBV infection

The study population was further stratified for a subgroup analysis, distinguishing CHB patients without cirrhosis or HCC (n = 549) from CHB patients with cirrhosis (n = 333) and CHB patients with HCC (n = 318) (Table 3). The S267F polymorphism was identified in 3.3% (n = 18) of patients with CHB only, 0.9% (n = 3) of patients with CHB and cirrhosis, and 3.5% (n = 11) of patients with CHB and HCC. The frequency of CT heterozygote was higher in CHB only patients than in cirrhotic patients with CHB. As shown in Table 3, the presence of S267F variant correlated significantly with a lower risk of cirrhosis development (OR 0.268, 95% CI 0.078–0.917, *P* = 0.036).

**Table 3:** Frequencies of S267F variant of NTCP in patients with chronic hepatitis B, cirrhosis, and HCC.

Polymorphism	CHB only	CHB + LC	CHB + HCC	CHB vs. LC		CHB vs. HCC		LC vs. HCC	
	n = 549	n = 333	n = 318	*P*	OR (95% CI)	*P*	OR (95% CI)	*P*	OR (95% CI)
S267F									
CC	531 (96.7)	330 (99.1)	307 (96.5)	1.000		1.000		1.000	
CT	18 (3.3)	3 (0.9)	11 (3.5)	0.036	0.268 (0.078–0.917)	0.887	0.946 (0.441–2.029)	0.037	0.254 (0.070–0.918)

Data are expressed as n (%). CHB, chronic hepatitis B; LC, liver cirrhosis, HCC, hepatocellular carcinoma; OR, odds ratio; 95% CI, 95% confidence interval.

### Correlation of S267F variant with HBV DNA levels in patients who did not receive antiviral therapy

According to the previous *in vitro* experiments^15^, the function of NTCP as a cellular receptor for HBV infection was substantially impaired by the S267F variant. This result suggests that the presence of this variant could lead to reduced HBV genome replication, the effect of which might be manifested as a lower viral DNA level. To address this possibility, the HBV DNA levels of treatment-naïve patients with and without S267F variant were compared (Table 4). Among the 32 CT heterozygote patients, 10 (31.3%) were treatment-naïve, and these individuals had a median DNA level of 2.0 log_10_ IU/mL (range, 1.08–4.66 IU/mL). In comparison, the median DNA level of the 477 treatment-naïve CC homozygote patients was 2.9 log_10_ IU/mL (range, 1.30–8.10, *P* = 0.179). Although the difference was not statistically significant, the observed reduction of HBV DNA level was consistent with the notion that the reduction of the viral entry may lead to the decreased viral load.

**Table 4:** HBV DNA levels in patients with and without S267F variant of NTCP.

	NTCP (wild type)	NTCP (S267F variant)	*P* value
HBV DNA level (log_10_IU/mL)	2.9 (1.30–8.10)	2.0 (1.08–4.66)	0.179

The patients for this analysis had no prior experience of antiviral therapy. Data are expressed as median (range). HBV, hepatitis B virus.

### Conserved preS1 sequence of HBV isolated from the CT heterozygote patients

**Figure 1:**
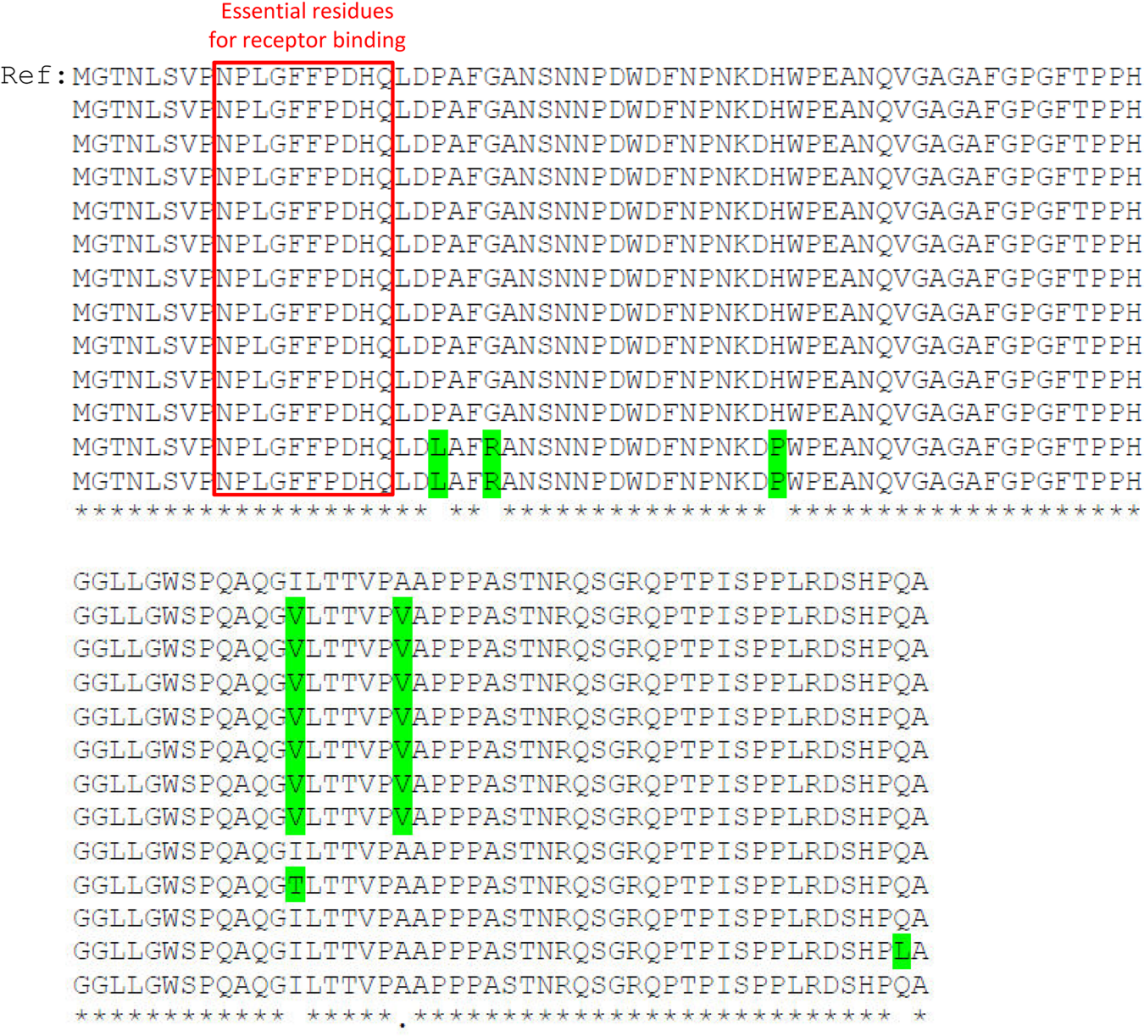
PreS1 domain sequence alignment. PreS1 amino acid sequence was determined from the viruses isolated from the CT heterozygote patients of NTCP and they were compared to that of the reference HBV sequence (GenBank AY247031.1). The essential residues for receptor binding were noted with a red rectangle. The residues different from the reference sequence were colored green.

Our data and the previous *in vitro* study^15^ demonstrated that the S267F variant negatively affects HBV infection. Although the CT heterozygote of NTCP may allow HBV infection, the infection could most likely be reduced compared to that by the CC wild-type homozygote. Since the entry of HBV into the hepatocyte requires interaction between NTCP and the large viral envelope glycoprotein via preS1 domain, the presence of S267F variant of NTCP might exert a selective pressure on the virus to cause compensatory mutation in the preS1 domain so that it can facilitate a more efficient interaction between NTCP and the preS1 domain. To examine this possibility, HBV viral DNAs were isolated from the patients who had the S267F variant of NTCP. Viral DNAs were amplified by PCR and the sequence of preS1 domain was determined by direct sequencing reactions. A total of 12 preS1 sequences were determined and compared to the reference preS1 sequence of genotype C HBV (Fig. 1). Despite several differences compared to the reference sequence, the residues essential for receptor interaction remained unaltered. This result suggests that if there is any selective pressure on the virus due to the presence of S267F variant it may not be strong enough to drive any viral evolution in the essential preS1 domain.

### Effect of ursodeoxycholic acid on hepatitis B virus infection

**Figure 2:**
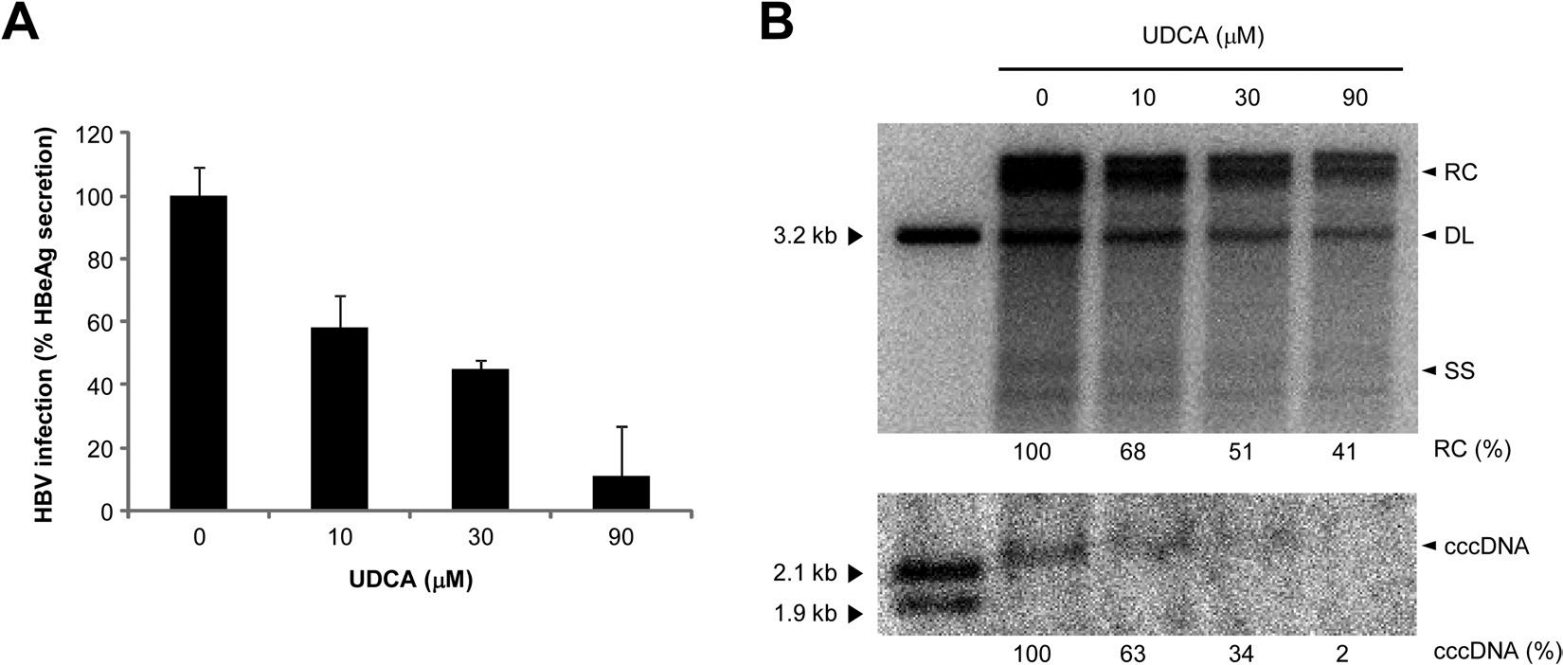
Effect of UDCA on HBV infection. To examine the effect of UDCA on HBV infection, HBV and UDCA were inoculated simultaneously to the HepG2-NTCP cells. For measurement of virus infection, ELISA for HBeAg (**A**) and Southern blot for HBV DNA replicative intermediates and cccDNA (**B**) were used. HBV infection decreased in a dose-dependent manner when the amount of UCDA increased. (RC, relaxed-circular; DL, duplex-linear; SS, single-stranded).

Prior to identification of NTCP as a cellular receptor for HBV infection, this protein had been known only as a bile acid transporter. Thus, one simple question arises regarding the dual roles of NTCP protein: whether NTCP can function simultaneously as HBV receptor and bile acid transporter. In other words, is the function as a viral receptor independent of that as a bile acid transporter? This question has already been addressed by Yan *et al*.^15^ and they demonstrated that the presence of several different bile acids inhibit HBV infection in *in vitro* cell culture setting. This is particularly interesting in that some CHB patients with high ALT levels are already being prescribed with UDCA and this prescription could suppress further HBV infection unintentionally by the competitive interaction of UDCA with NTCP. To address whether UDCA indeed suppresses HBV infection, we inoculated HBV to HepG2-NTCP cells^16^ in the presence of differing amounts of UDCA. The measurement of HBeAg by ELISA indicated that UDCA inhibited HBV infection in a dose-dependent manner (Fig. 2A). Quantification of the HBV DNA replicative intermediates and cccDNA by Southern blot analysis also corroborated this conclusion (Fig. 2B). Thus, these results suggest that UDCA could be used as a potential antiviral agent against HBV infection.

## Discussion

NTCP plays an important physiological role in both enterohepatic circulation of bile acids and hepatocyte function^17^. Although NTCP had been known as a bile acid transporter for a long time^13,14^, a completely different function as a cell surface receptor for both HBV and HDV infection was recently uncovered^7,10^. Interestingly, the functional determinants as HBV receptor and bile acid transporter are at least in part shared on the same NTCP protein^15,18^. Thus, genetic variations of NTCP could potentially have effects on both HBV entry and bile acid uptake. To date, six SNPs have been identified in NTCP^14^ and some of these SNPs are found in specific ethnic backgrounds. Among them, the S267F substitution in exon 4 of NTCP gene affects both HBV entry and bile acid uptake and has been identified only in East Asian people^15,19^. However, the effect of this specific substitution on HBV infection and subsequent liver diseases was not completely understood in patients with chronic hepatitis B.

To address this question, we analyzed a total of 1,376 samples from CHB patients and controls. Importantly, our results showed that people with the S267F variant of NTCP were less susceptible to HBV infection and that the CHB patients with S267F variant had a lower risk of developing cirrhosis. Cirrhosis develops as a result of inflammation and fibrosis in patients with CHB and an increased HBV DNA level is one of the most important risk factors for cirrhosis development^2^. Since the S267F variant of NTCP is associated with lower HBV DNA levels (Table 4), the risk of cirrhosis development could be reduced in the patients with the S267F variant. However, we did not find any significant, direct association between the S267F variant and HCC development. We speculate that carcinogenesis occurs from many different mechanisms and indeed cirrhosis itself is the strongest factor associated with HCC development.

We also sequenced the preS1 domain of HBV L protein from the S267F variant-containing samples to see whether the presence of S267F variant exerted a selective pressure on the virus that is strong enough to drive any viral evolution in the preS1 domain. However, we could not find any significant sequence variation, especially in the essential residues for NTCP interaction. Perhaps, the pressure was not strong enough to drive evolution or the presence of the other “C” wild-type allele might compensate for the defect of the “T” allele. In fact, a previous *in vitro* investigation of “CT” heterozygote by co-transfecting the wild-type and S267F-contining NTCP expression plasmids in a 1:1 ratio displayed ~ 70% receptor activity^15^ and this level of efficiency might be sufficient for HBV infection.

Since the two distinct functions of NTCP are not completely independent from each other, HBV infection can be competitively inhibited by the treatment with bile acids^15^ and we observed a similar inhibitory effect of UDCA on HBV infection (Fig. 2). UDCA is known to reduce ALT levels in patients with chronic liver diseases^20^ and the patients with high ALT levels are already being prescribed with UDCA. Thus, taking UDCA can potentially reduce further HBV infection by competitive inhibition of HBV entry into the uninfected hepatocytes. However, the concentration of UDCA that we used in our experiment (0–90 μM) might not be identical to the ones in real clinical settings, in which standard doses of UDCA treatment are within the range of 12–15 mg/kg body weight^21^. Further studies are required regarding the influence of UDCA in the course of HBV infection and subsequent pathogenesis.

While we were conducting this investigation, three other studies on Chinese Han and Taiwanese people were reported regarding the association between NTCP polymorphism and the clinical characteristics of CHB. Since all the outcomes from these are not completely identical, we summarized and compared the results of these studies including ours in Table 5. All studies were performed for East Asian people since the S267F variant of NTCP is found in this ethnic group. In summary, there are some similarities and differences among these studies. However, a few conclusions could be drawn from these related studies: (1) People who have the S267F variant of NTCP are less susceptible to HBV infection. (2) The S267F variant of NTCP is associated with a lower risk of subsequent liver diseases including acute-on-chronic liver failure, cirrhosis or HCC. Only the first study^19^ presented conflicting data about susceptibility to HBV infection and subsequent pathogenesis perhaps due to the relatively small number of samples they analyzed.

**Table 5:** Summary and comparison of the studies on the association between NTCP polymorphism and chronic hepatitis B.

Authors	Li *et al*.^19^	Peng *et al*.^24^	Hu *et al*.^25^	This Study
Ethnic background	Chinese Han	Chinese Han	Taiwanese	Korean
Study population	n = 244 (HBV patients) n = 76 (HBV infection resolvers)n = 113 (healthy controls)	n = 1,899 (patients with CHB) n = 1,828 (healthy individuals)	n = 3,801 (patients with CHB) n = 3,801 (HBsAg-seronegative controls)	n = 1,200 (patients with CHB) n = 176 (controls)
Frequencies of S267F variant (CT and TT)	11.9% in patients 5.3% in resolvers 4.4% in controls	8.1% in patients 20.4% in controls	18.5% in patients 17.3% in controls	2.7% in patients 5.7% in controls
Effect of S267F variant on HBV infection	HBV infection ⇑	HBV infection ⇓	HBV infection ⇓	HBV infection ⇓
Effect of S267F variant on liver diseases	No significant association between S267F and cirrhosis, HCC	ACLF ⇓	Cirrhosis ⇓, HCC ⇓	Cirrhosis ⇓

CHB, chronic hepatitis B; HBV, hepatitis B virus; HCC, hepatocellular carcinoma; ACLF, acute-on-chronic liver failure; ⇑, increase; ⇓, decrease.

Our study had a few limitations. First, the allele frequency of S267F variant was low and we did not perform additional population stratification. However, we confirmed that the frequency of S267F variant in our study was almost identical to that of the previous report (5.0%). Subsequent studies are warranted including more matched controls. Second, the number of controls in this study was small. However, we confirmed that there was no statistical difference in age and gender between the two groups before analysis. CHB only group in this study consisted of patients with relatively similar age, gender, lower HBV DNA level (median 2.4 log_10_IU/mL) and no cirrhosis.

Studying the S267F variant of NTCP and its effect on HBV infection and pathogenesis is possible only in East Asian countries. In this regard, the results from the three different countries on three different ethnic groups (Chinese Han, Taiwanese and Korean) are important in that they provide a comprehensive view of this unique SNP and its clinical significance. The information obtained from these studies might help predict the occurrence of advanced liver diseases due to HBV infection and identify individuals of high or low risk of further complications.

## Methods

### Patients

This study included 1,200 patients with CHB and 176 controls. The CHB patients were defined as HBsAg-positive individuals for at least 6 months and the controls were defined as HBsAg-seronegative individuals who had no previous HBV immunization or were uncertain about vaccination history. All participants were recruited at Severance Hospital, Yonsei University College of Medicine, Seoul, Korea. Of the 1,200 patients with CHB, 333 were diagnosed with cirrhosis and 318 were diagnosed with HCC. Patients infected with other hepatitis viruses (with the exception of hepatitis C virus), autoimmune disorders, and other non-HBV diseases were excluded. Blood samples and clinical data were collected from SOLID-CORE (Severance Hospital Liver Disease-Cohort Registry) system. Written informed consent was obtained from the patients or responsible family members. This study was approved by the independent Institutional Review Board of Severance Hospital and conformed to the ethical guidelines of the 1975 Helsinki Declaration.

### Chromosomal DNA isolation and genotyping of S267F polymorphism

Genomic DNA was isolated from whole blood using the MiniBEST Universal Genomic DNA Extraction Kit Ver.5.0 (Takara) according to the manufacturer’s instruction. The S267F polymorphism of NTCP was determined using the polymerase chain reaction–restriction fragment length polymorphism (PCR-RFLP) method as described^19^.

### Clinical evaluation

At baseline, routine blood chemistry parameters, serum HBV DNA levels, and other serologic viral markers were assessed. HBsAg, HBeAg, and anti-HBe were measured using ELISA (Abbott Laboratories). Serum HBV DNA levels were quantified using a commercially available real-time polymerase chain reaction (PCR) assay (COBAS AmpliPrep-COBAS TaqMan HBV test, Roche) with a linear detection range of 20–170,000,000 IU/mL. Serum alanine aminotransferase (ALT) levels were measured using standard laboratory procedures with the upper limit of normal set at 40 IU/L. If histologic information was not available, clinically diagnosed cirrhosis was defined as follows: (1) platelet count <100,000/μL and ultrasonographic findings suggestive of cirrhosis, including a blunted, nodular liver edge accompanied by splenomegaly (>12 cm); or (2) esophageal or gastric varices^22^. During follow-up, all patients underwent periodic surveillance with ultrasonography and laboratory workups, including determination of α-fetoprotein levels, at 3 or 6 month intervals.

### HBV infection in the presence of ursodeoxycholic acid

HepG2-NTCP cells, which stably express NTCP protein, and the procedure for HBV production were described^16^. For infection, HepG2-NTCP cells were seeded in 48-well plates at a density of 7.5 × 10^4^ cells/well. Twenty four hours later, the cells were inoculated with HBV in the presence of 4% PEG 8000 and ursodeoxycholic acid (UDCA) (0, 10, 30 or 90 μM) at 37 °C for 24 h. The infected cells were washed and then maintained in the fresh culture medium in the presence of 2.5% DMSO. Culture supernatants were collected regularly and a commercial ELISA kit was used to measure secreted HBeAg levels (Wantai). For Southern blot analysis, HBV DNA replicative intermediates and cccDNA were prepared as described previously^16^.

### Viral DNA isolation and preS1 sequencing

Viral DNA was isolated from patients’ sera using QIAamp MinElute Virus Spin Kit (Qiagen). A DNA fragment containing preS1 was specifically amplified by PCR from the isolated viral DNA using the following primers; 5′- CATACTCTGTGGAAGGCTGG -3′ (forward) and 5′- TGAGGCAGTAGTCGGAACAG -3′ (reverse). The amplified DNA was then sequenced and compared to the reference preS1 sequence of genotype C HBV (GenBank AY247031.1).

### Statistical analysis

Data are expressed as mean ± SD, median (range), or n (%), as appropriate. Differences among continuous variables were examined for statistical significance by Student’s *t-*test (or Mann-Whitney test, if appropriate). Categorical variables were analyzed by chi-square test (or Fisher’s exact test, if appropriate). The Cox proportional hazards model was used for multivariate analyses. Hardy-Weinberg equilibrium (HWE) was tested using the Online Encyclopedia for Genetic Epidemiology HWE tool (OEGE)^23^. Polymorphism association was analyzed with Chi-square (X^2^) test. All statistical analyses were performed using the Statistical Package for the Social Sciences (SPSS version 20.0) and SAS (SAS version 9.2). A value of *P* < 0.05 was considered to indicate statistical significance.
